# Limpet (*Scutellastra cochlear*) Recovered From Some Estuaries in the Eastern Cape Province, South Africa Act as Reservoirs of Pathogenic *Vibrio* Species

**DOI:** 10.3389/fpubh.2018.00237

**Published:** 2018-08-31

**Authors:** Oluwatayo E. Abioye, Anthony I. Okoh

**Affiliations:** ^1^SAMRC Microbial Water Quality Monitoring Centre, University of Fort Hare, Alice, South Africa; ^2^Applied and Environmental Microbiology Research Group, Department Of Biochemistry and Microbiology, University of Fort Hare, Alice, South Africa; ^3^Department of Microbiology, Obafemi Awolowo University, Ile-Ife, Nigeria

**Keywords:** public-health, MPN-PCR, reservoir types, *Vibrio* species, limpet, prevalence, transient reservior, maintanance reservoir

## Abstract

Limpet (*Scutellastra cochlear*) serves as seafood recipe and an important member of the aquatic food chain. It is an abundant mollusc in some aquatic environment in South Africa. In this study, we investigated the potential of the molluscs harvested from the Buffalo, Swartkops, and Kowie estuaries in the Eastern Cape Province, South Africa to serve as transient or maintenance reservoir of *Vibrio* species. The mollusc and source water samples were collected monthly from the rivers between December 2016 and November 2017. The reservoir category of the limpet samples recovered was determined by employing the combination of MPN-PCR method and statistical analysis (comparison of mean and proportion tests). The densities of *Vibrio* spp. in limpet and their source water samples were determined using MPN-PCR methods. Presumptive isolates were recovered by processing the samples with thiosulfate-citrate-bile salts-sucrose agar and where necessary, samples were enriched with alkaline peptone water. The presumptive isolates were identified using PCR methods with emphasis on six *Vibrio* species of public health importance. *Vibrio* spp. were detected in all the limpet samples but not in all the water samples. The densities of *Vibrio* spp. in the limpet samples were more than the densities of *Vibrio* species in their source water and these were significant at *P* < 0.05. In like manner, five out of the six key *Vibrio* pathogens targeted in this study were more prevalent in limpet samples than in source water samples. Based on our findings, we concluded that our method though could be improved on, is efficient for the determination of reservoir types of bacterial-carrying organisms. We also concluded that the limpet found in the estuaries are not just a transient but a maintenance reservoir of *Vibrio* spp. which could cause vibrio-related infections.

## Introduction

The factors responsible for the persistence of cholera and cholera-like infections in the world despite the effort put in place by international public health stakeholders such as The Centre for Disease Control and Prevention (CDC) and World Health Organization (WHO) have been debated over the years. One of the factors that is on for debate by public health stakeholders is the possibility of the presence of reservoirs for *Vibrio cholerae* and other pathogenic vibrios in the environment. A year (2012–2013) survey on toxigenic *Vibrio cholera* O1 in Haiti by Alam et al. (1) suggested that the persistence of cholera is due to the existence of environmental reservoir. This finding was challenged by CDC (2) in a letter addressed to the authors of the study on the basis that the epidemiological data supplied were not explicit enough. It was pointed out that the work of Alam et al. (1) was carried out during an ongoing cholera epidemic and heavy rainfall which proposed the possibility of contamination of waterbody by open-air defecation especially by individuals with the disease. Thus, Stanislas and Renaud (2) pointed out the need to carry out this kind of investigation when there is no outbreak in other to confirm if *V. cholerae* reservoirs actually exist in the environment or if the so-called reservoirs are just transient vectors. The outbreak of vibrio infections such as cholera is believed to be preceded by environmental bloom and subsequent spilling of the pathogens into human population via vehicles and vectors of infections such as water and aquatic animals (1, 3). The ability of these pathogens to bloom in the environment could be as a result of the existence of reservoirs which concentrate and shield them against unfavorable conditions. There are several studies that have confirmed the presence of pathogenic *Vibrio* spp. in abiotic and biotic components of the aquatic environments. The notable studies are those that reported foodborne infections outbreaks caused by seafood. The seafood in this category can be broadly classified into three and these are fishes, molluscs, and crustaceans. Some of the molluscs that have been linked to vibrio infections include bivalves such as mussels, oysters, scallops, and clams while some of the crustaceans that have been implicated in vibrio infections are crabs, shrimps, and prawns. Likewise, the presences of the pathogens in both freshwater and saltwater have been reported. Studies on the isolation of these pathogenic vibrios from molluscs and crustaceans have been reported from different geographical locations of the world. These reports include those from Korea, Spain, Poland, Morroco, Egypt (4–8). The various studies mentioned earlier have been able to prove that aquatic animals have the ability to harbor pathogenic *Vibrio* species but none have been able to experimentally proven if these seafood are just transient or concentrating carriers. Thus this study statistically compared the densities of total halophiles and *Vibrio* species in limpet (*Scutellastra cochlear*) samples and their source water and the prevalence of the targeted six key *Vibrio* pathogens in limpet and their source water in an attempt to put forward a method that could be used to confirm if an organism is a transient or a concentrating reservoir of bacteria of interest which in this case is *Vibrio* spp. The *Vibrio* species isolated were further delineated into the key six vibrio pathogens (*Vibrio cholerae, Vibrio mimicus, Vibrio fluvialis, Vibrio alginolyticus, Vibrio vulnificus*, and *Vibrio parahaemolyticus*) to substantiate the findings of this study.

Limpet (*Scutellastra cochlear*), a native of South Africa (9, 10) which serves as seafood recipe and an important member of the aquatic food chain is an abundant mollusc in some aquatic environment in South Africa. It has been reported that limpet should be prepared like other gastropods e.g., abalone to achieve tasty recipe. The cooking is to be done at high temperature for say 30 s or at low temperature for a longer period (11). Southern Africa has been recognized as the world's foremost biodiversity and biomass hotspot for this mollusc. The density of the organism per square land mass of some of the aquatic environment in Southern Africa is as high as 2,600 (12). Limpet serves an important ecological role in the aquatic food chain and as earlier mentioned a food recipe for human (13). Although the prevalence of *Vibrio* spp. in several molluscs that are important to humans have been investigated, unfortunately, there is a dearth of information as regards the relationship between *Vibrio* spp. and Limpet. Thus, we assessed the potential of the limpets recovered from the Buffalo, Swartkops, and Kowie estuaries in the Eastern Cape Province, South Africa as a reservoir of *Vibrio* species with emphasis on six key potential pathogenic ones. The reservoir type of the mollusc was also determined.

## Materials and methods

### Sampling sites and sample collections

The coordinates of the sampling sites and their peculiar features that qualified them for inclusion in this study are as articulated in Table 1 below.

**Table 1:** Characteristics of sampling sites and their coordinates.

**Sites**	**Codes**	**Municipalities**	**Coordinates**	**Peculiarities**
Buffalo estuarine site	EL5	Buffalo City Metropolitan	33°01′21.8^′′^S 27°53′40.4^′′^E	An estuarine located along Buffalo river. It receives industrial effluents and it is used for fishing activities.
Kowie Estuarine	PA7	Sarah Baartman	33°35′12.1^′′^S 26°52′54.3^′′^E	It is located in Port Alfred. It is used for recreational activities e.g., leisure fishing and swimming.
Swartkops Estuarine	SKR	Nelson Mandela Bay	33°51′31.1^′′^S 25°35′56.4^′′^E	It is an estuarine located in Port Elizabeth. Serious fishing and recreational activates occur at this site. It is surrounded by a number of companies and wastewater treatment plants. The water smells awful and this suggests that the surrounding companies might be discharging poorly treated effluents into the river

All the Limpet samples used in this study were purchased from fishermen, transferred into a sterile stomacher bags and transported on ice to the laboratory of the Applied and Environmental Microbiology Research Group (AEMREG), University of Fort Hare, Alice, South Africa. In like manner, water resources from where the limpet samples were recovered were also sampled and all samples were processed within 6 h of collection. Limpet samples were identified using limpet atlas (9, 14–16) and samples (water and limpet) were collected per month based on availability of limpet between December 2016 and November 2017.

### Determination of total *vibrio* spp. density in limpet (*scutellastra cochlear*) and water samples

Total vibrio density in the limpet and water samples were determined using 3-tubes by 5-dilutions end-point MPN-PCR techniques as described by Noorlis et al. (17), Copin et al. (18), and Ramos et al. (19). The MPN-PCR method is as reported by Tunung et al. (20). Briefly, 5–12 pieces each of molluscs limpet, were aseptically pooled together in a sterile stomacher bags and pummeled using stomacher machine (BagMixer 400, Interscience). A 10 g portion of the pummeled samples was afterwards added to a separate conical flask containing 90 ml sterile 3% NaClTryptic Soy broth to form initial homogenate which is the first dilution (10^1^). The remaining four dilutions were prepared from the initial homogenate using alkaline peptone water (APW) as the diluent and 1 ml aliquot from each of the dilutions was inoculated into a tube containing fresh sterile 9 ml of APW. The setups were done in triplicates and resulting inoculated tubes were incubated at 37°C for 24 h. The water samples were analyzed in a similar manner except that the first dilution was prepared using double strength APW. After the incubation period, turbid tubes were recorded and corresponding MPN values were extrapolated from BAM-MPN excel spreadsheet. The extrapolated values were taken as the total halophile density. Afterwards, DNA extracted from 1 ml aliquot from each turbid tube was subjected to PCR following the procedure stated in sections Genomic DNA Extraction and Molecular Confirmation of Presumptive *Vibrio* isolates below, for the detection of 16S rRNA gene region specific for *Vibrio* spp. Turbid tubes that were positive for *Vibrio* spp. were noted and corresponding MPN values were extrapolated as earlier stated. The MPN value at this point was taken as total vibrio density. Total halophile's density and *Vibrio* species density of water samples from which limpet were recovered where also determined using MPN-PCR method as described earlier. This was done in other to statistically compare the total *Vibrio* species load in aquatic animals and their source water without introducing bias.

### Enrichment of samples

In other to achieve an optimum isolation of the targeted *Vibrio* spp. 10 g sample each of the remaining pummeled limpet samples in section Determination of Total *Vibrio* spp. Density in Limpet (*Scutellastra cochlear*) and Water Samples above and 10 mL of source water samples were also inoculated into 90 ml sterile APW. The setups were agitated gently for 1–2 min and incubated at 37°C for 24 h (21). After incubation period, a loop-full was carefully taken from the pellicle formed on the surface of the APW and streaked on fresh sterile TCBS plates.

### Isolation of presumptive *vibrio* spp. from limpet samples

About 5 to 10 distinct yellow and green colonies from section Enrichment of Samples were carefully picked and purified on fresh sterile TCBS followed by nutrient agar plates that contained 3% NaCl. The recovered isolates were taken as presumptive *Vibrio* spp. The purified presumptive *Vibrio* spp. were grown overnight at 37°C on sterile nutrient agar plates and 20% glycerol stock of the pure isolates were then prepared and stored in −80°C for further analysis.

### Molecular identification of presumptive *vibrio* isolates

#### Genomic DNA extraction

The DNA extraction was carried out by following the boiling method procedure described by Maugeri et al. (22). An 18 h old culture incubated at 37°C was prepared on sterile nutrient agar plate from the stock culture of presumptive *Vibrio* isolates stored at −80°C and genomic DNA was extracted using boiling method. Single colonies were picked from the nutrient agar plates, suspended in a microfuge tube containing 200 μl of sterile distilled water and vortexed for even distribution of cells in the diluent. The cells were then lysed using AccuBlock (Digital dry bath, Labnet) for a period of 15 min at 100°C. The resulting solution was afterwards centrifuged at 110,000x g for 2 min using a MiniSpin microcentrifuge to remove the cell debris. The supernatant which is the cell lysate was then used as DNA templates in PCR assay.

#### Molecular confirmation of presumptive *vibrio* isolates

The confirmation of presumptive *Vibrio* isolates was done using *Vibrio* genus-specific primers that target the variable regions around position 700 and 1325 within 16S rRNA gene in a PCR assay. The cell lysate (5 μL) prepared in section Genomic DNA Extraction above was used in a 25 μl reaction containing 12.5 μl of one *Taq* 2X Master Mix Standard Buffer (*BioLabs*, UK), 1 μl of 10 μM of forward and reverse primer and 5.5 μl of nuclease-free water. The thermal cycler profile was a single enzyme activation for 15 min at 93°C followed by 35 cycles at 92°C for 40 s, 57°C for 1 min and 72°C for 1.5 min and a final extension at 75°C for 7 min. *V. alginolyticus* DSM 2171 was used as the positive control. The primer sequences, source of primer and expected amplicon size is presented in Table 2.

**Table 2:** Primers for confirmation into *Vibrio* genus and delineating into pathogenic species.

**Specie**	**Sequence**	**Size bp**	**References**
*Vibrio* spp.	F: CGG TGA AAT GCG TAG AGA T R: TTA CTA GCG ATT CCG AGT TC	663	(23, 24).
*V. cholerae*	F: CAC CAA GAA GGT GAC TTT ATT GTG R: GGT TTG TCG AAT TAG CTT CAC C	304	(25, 26).
*V. parahaemolyticus*	F: GCA GCT GAT CAA AAC GTT GAG T R: ATT ATC GAT CGT GCC ACT CAC	897	(24, 27).
*V. vulnificus*	F: GTC TTA AAG CGG TTG CTG C R: CGC TTC AAG TGC TGG TAG AAG	410	(24, 28).
*V. Fluvialis*	F: GAC CAG GGC TTT GAG GTG GAC GAC R: AGG ATA CGG CAC TTG AGT AAG ACTC	217	(24, 29, 30)
*V. Mimicus*	F: GGTAGCCATCAGTCTTATCACG R: ATCGTGTCCCAATACTTCACCG	390	(31–34)
*V. alginolyticus*	F: GAGAACCCGACAGAAGCGAAG R: CCTAGTGCGGTGATCAGTGTTG	337	(35, 36)

#### Speciation of confirmed *vibrio* species

The confirmed *Vibrio* species were delineated into the six *Vibrio* spp. targeted in this study using species-specific primers. The primer sets that target conserved region of *fla E, GroEl, ToxR, OmpW, vhm*, and *GyrB* genes were used in PCR reactions to confirm *Vibrio* spp. as *V. parahaemolyticus, V. vulnificus, V. fluvialis, V. cholerae, V. mimicus, and V. alginolyticus* respectively. Duplex PCR protocol was developed for simultaneous identification of *V. cholerae* and *V. mimicus* while triplex PCR protocol was developed for simultaneous identification of *V. vulnificus, V. fluvialis*, and *V. alginolyticus* following the multiplex PCR protocol guide of Sint et al. (37) Briefly, DNA templates were extracted as stated in section Genomic DNA Extraction from positive controls. The duplex PCR reaction was made up of 12.5 μl *Taq* 2X Master Mix Standard Buffer (*BioLabs*, UK), 1 μl (0.2 μM) of forward and reverse primer set specific for each of the targeted species, 2.5 μl each of their DNA templates and 3.5 μl of nuclease-free water. The reaction mixture for the triplex PCR was made up of 12.5 μl *Taq* 2X Master Mix Standard Buffer (*BioLabs*, UK), 0.5 μl (0.2 μM) per forward and reverse primer set for each targeted species, 2.5 μl each of their DNA templates and 2 μl of nuclease-free water. The multiplex system was then tested in a gradient PCR with single extracts and a mix of all targeted species to determine the optimal annealing temperature using positive controls. The procedure was repeated severally to ascertain the reproducibility of the result. The multiplex PCR amplification efficiency, sensitivity, and primer specificity were tested following (37) procedure. Molecular Identification of *V. parahaemolyticus* was carried out using simplex PCR protocol in section Molecular Confirmation of Presumptive *Vibrio* isolates except that the annealing temperature employed was 64°C. The positive controls used were *V. parahaemolyticus* (DSM 10027), *V. vulnificus* (DSM 10143), *V. fluvialis* (DSM 19283), *V. mimicus* (DSM 19130), *and V. alginolyticus* (DSM 19130) and one locally isolated *V. cholerae*. The annealing temperatures selected for duplex and triplex PCR protocols, based on the gradient PCR outcomes, were 54.5°C and 66.3°C, respectively. These optimized conditions were used for the speciation of the confirmed *Vibrio* spp. into the six *Vibrio* species we target in this study. *E. coli ATCC 35150* was used as the internal control for all PCR reactions. The primer sequences, the source of primer and expected amplicons sizes are presented in Table 2.

### Statistical analysis

The mean halophile and vibrio densities in limpet and source water samples were statistically compared using One-Way Analysis of Variance (ANOVA). Fisher's Least Significant Difference (LSD) *post-hoc* test was used to further compare and explore the significance of mean differences of the halophiles and *Vibrio* spp. densities in limpet and source water samples. The Statistical Package for Social Sciences (SPSS) version 20 was employed for the analysis. Also, the significant differences between the prevalence of each of the vibrio species we considered in this study amidst the population of confirmed *Vibrio* species isolated from limpet and source water samples were computed using 5000 Monte Carlo simulations tool of the XLSTAT evaluation 2018.1 software. The critical *P* value was set at 0.05.

## Result

### Total halophiles and total *vibrio* species densities in limpet and source water samples

**Figure 1:**
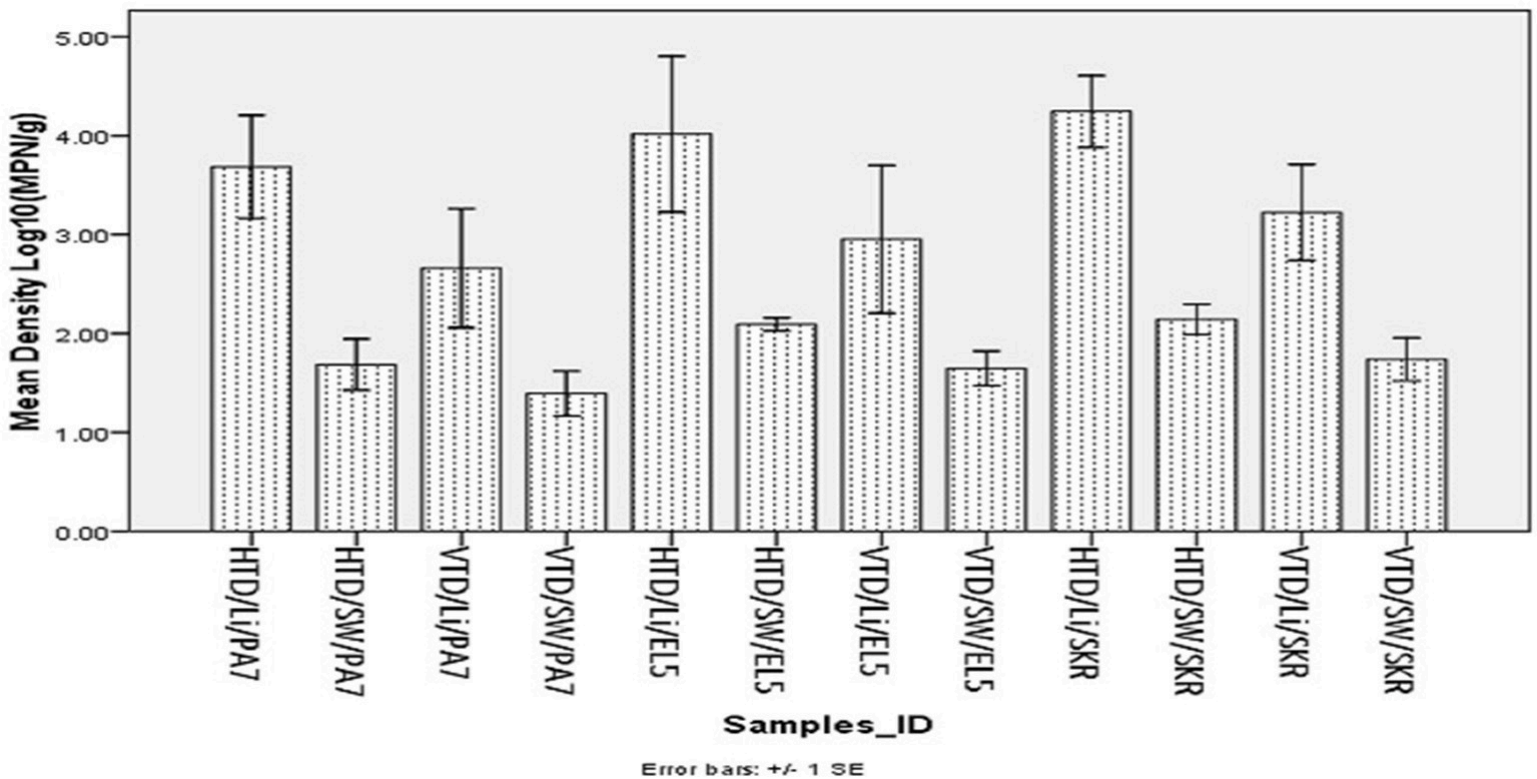
Mean density of halophile and *Vibrio* species in limpet and source water. Key: HTD, halophile total density; VTD, *Vibrio* spp. total density; Li, limpet sample; SW, limpet source water samples; PA7, Kowie estuarine; EL5, Buffalo estuarine; SKR, swartkops estuarine.

The mean total halophile and total *Vibrio* species densities observed for limpet samples and source water are presented in Figure 1 below while the significant levels (*P values*) for the statistical comparison of the mean halophiles and *Vibrio* species densities in the limpet samples and source water are presented in Table 3.

**Table 3:** Significant levels of the statistical comparison of halophiles and *Vibrio* spp. densities in limpet samples and source water samples.

**Sampling sites**	**Total halophiles**		**Sampling sites**	**Total** ***Vibrio*** **spp. densities**	
	**Sample types**	***P-values***		**Sample types**	***P-values***
PA7	Limpet	0.001^*^	PA7	Limpet	0.026^*^
	Source water			Source water	
EL5	Limpet	0.038^*^	EL5	Limpet	0.154
	Source water			Source water	
SKR	Limpet	< 0.001^*^	SKR	Limpet	0.04^*^
	Source water			Source water	

* *, significant difference*.

The mean of total halophile density in limpet samples ranges from 3.69 ± 1.48 Log_10_ (MPN/g) in samples from PA7 to 4.25 ± 1.15 Log_10_ (MPN/g) in samples from SKR. In the limpet source water samples, the mean halophile density ranged between 1.69 ± 0.73 Log_10_ (MPN/g) in samples from PA7 and 2.14 ± 0.48 Log_10_ (MPN/g) in samples from SKR. The ranges of the mean of the total *Vibrio* species density in limpet and source water samples were 2.66 ± 1.7 Log_10_ (MPN/g) (limpet samples from PA7) to 3.22 ± 1.54 Log_10_ (MPN/g) (limpet samples from SKR) and 1.39 ± 0.64 Log_10_ (MPN/g) (source water samples from PA7) to 1.74 ± 0.69 Log_10_ (MPN/g) (source water samples from SKR) respectively. Of the samples from the three sites studied, lowest mean of the total halophile densities and mean of the total *Vibrio* species densities were observed in PA7 samples while the highest were observed in the samples collected at site SKR. The relatively high halophile and *Vibrio* species densities observed at site SKR could be as a result of high pollution observed at this site throughout the sampling period.

The densities of both halophiles and *Vibrio* species in limpet samples were consistently higher than that observed in sources water. This observation was statistically significant at *P* < 0.05 for all comparisons except for the densities of *Vibrio* species in limpet and source water samples from site EL5.

### Pooled prevalence of the six targeted *vibrio* spp. in limpet and sources water samples from site PA7, EL5, and Skr

**Figure 2:**
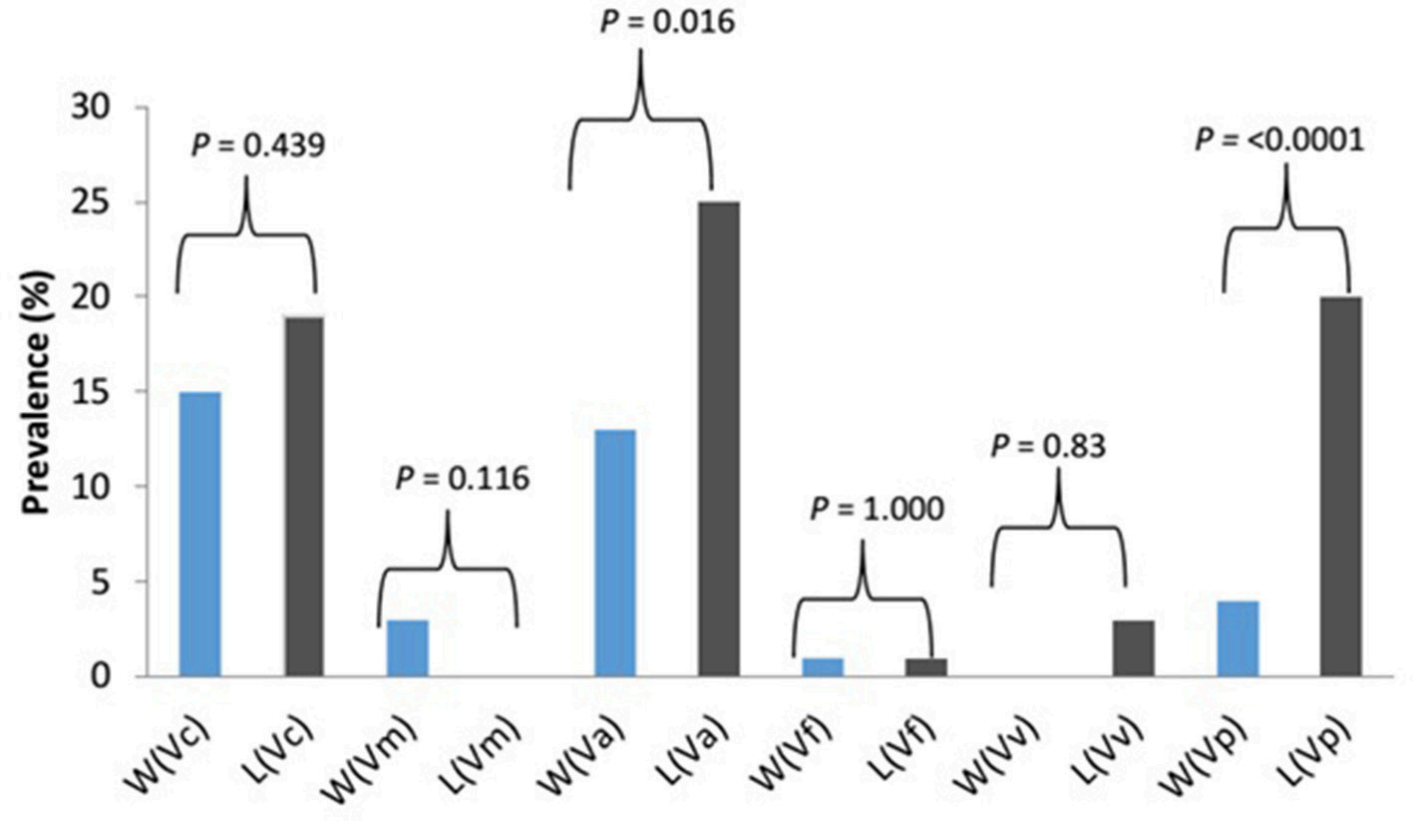
Prevalence and significant differences of six key vibrio pathogens in *Vibrio* species isolated from limpet and water samples. Key: W, water; L, limbet; Vc, *V. cholerae*; Vm, *V. mimicus*; Vf, *V. fluvialis*; Va, *V. alginolyticus*; Vv, *V. vulnificus*; Vp, *V. parahaemolyticus*; blue bars, prevalence in source water samples; black bars, prevalence in limbet samples.

The prevalence of *Vibrio* spp. and each of the six targeted species in the presumptive *Vibrio* isolates recovered from all limpet samples (*n* = 197) and water samples (*n* = 193) is presented in Figure 2. The overall prevalence of *Vibrio* spp. was 84% (166/197) and 81% (156/193) in limpet and source water samples, respectively. The prevalence of each of the targeted species amidst the confirmed *Vibrio* spp. (*n* = 166) isolated from limpet samples was 19% (32/166), 0% (0/166), 25% (42/166), 1% (2/166), 3% (5/166), and 20% (33/166) for *V. cholerae, V. mimicus, V. alginolyticus, V. fluvialis, V. vulnificus*, and *V. parahaemolyticus* while the prevalence of each of the targeted species amidst the confirmed *Vibrio* spp. (*n* = 156) isolated from source water samples was 15% (24/156), 3% (4/156), 13% (21/156), 1% (2/156), 0% (0/161), and 4% (6/161) for *V. cholerae, V. mimicus, V. alginolyticus, V. fluvialis, V. vulnificus*, and *V. parahaemolyticus* respectively. The prevalence of *V. alginolyticus*, and *V. parahaemolyticus* in limpet and source water samples were significantly different (Figure 2) but the prevalence of the remaining four of the six targeted *Vibrio* species in the two samples were not significantly different at a critical *P* value of 0.05 (Figure 2). The distribution of the six *Vibrio* species targeted in this study per season is as presented in Table 4. The table showed that greater percentage of the *Vibrio* species were isolated during the warm period of our sampling regime.

**Table 4:** Distribution of targeted *Vibrio* species in limpet and source water per season.

**Season**	**Source water**						**Limpet**					
	**Vc (*n)***	**Vm (*n*)**	**Vf (*n*)**	**Va (*n*)**	**Vv (*n*)**	**Vp (*n*)**	**Vc (*n*)**	**Vm (*n*)**	**Vf (*n*)**	**Va *(n)***	**Vv (*n*)**	**Vp (*n*)**
**Summer** (warm months)	16	3	1	13	–	4	5	–	–	6	1	6
**Autumn** (warm months)	5	–	–	8	–	2	27	–	2	28	–	20
**% Distribution** (warm months)	21/24 (88%)	3/4 (75%)	1/2 (50%)	21/21 (100%)	–	6/6 (100%)	32/32 (100%)	–	2/2 (100%)	34/42 (81%)	1/5 (20%)	26/33 (79%)
**Winter** (cool months)	3	1	1	–	–	–	–	–	–	7	4	6
**Spring** (cool months)	–	–	–	–	–	–	–	–	–	1	–	1
**% Distribution** (cool months)	3/24 (12%)	1/4 (25%)	1/2 (50%)	–	–	–	–	–	–	12/42 (19%)	4/5 (80%)	7/33 (21%)
**Total**	24	4	2	21	0	6	32	–	2	42	5	33

*Vc, V. cholerae; Vm, V. mimicus; Vf, V. fluvialis; Va, V. alginolyticus; Vv, V. vulnificus; Vp, V. parahaemolyticus; -, zero; n, number of isolate*.

**Figure 3:**
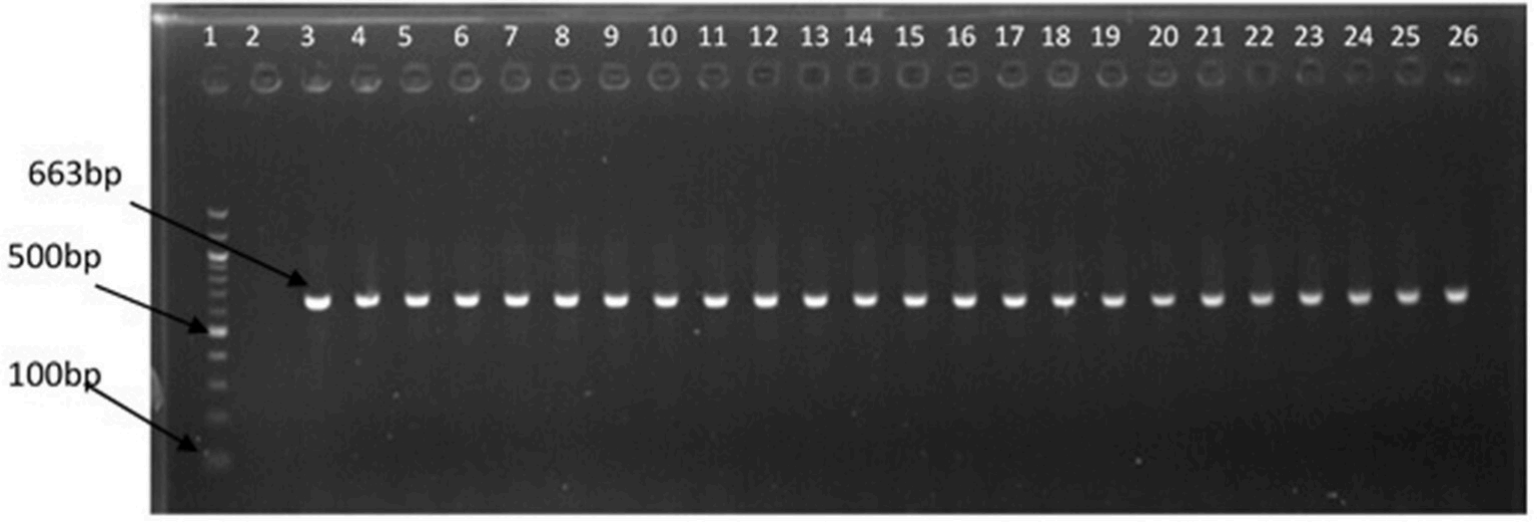
PCR products of the amplification of *Vibrio* specific region of 16S rRNA gene. Lane 1, molecular weight marker (100 bp); lane 2, negative control; lane 3, positive control (DSM 19130); lanes 4–26, positive isolates.

**Figure 4:**
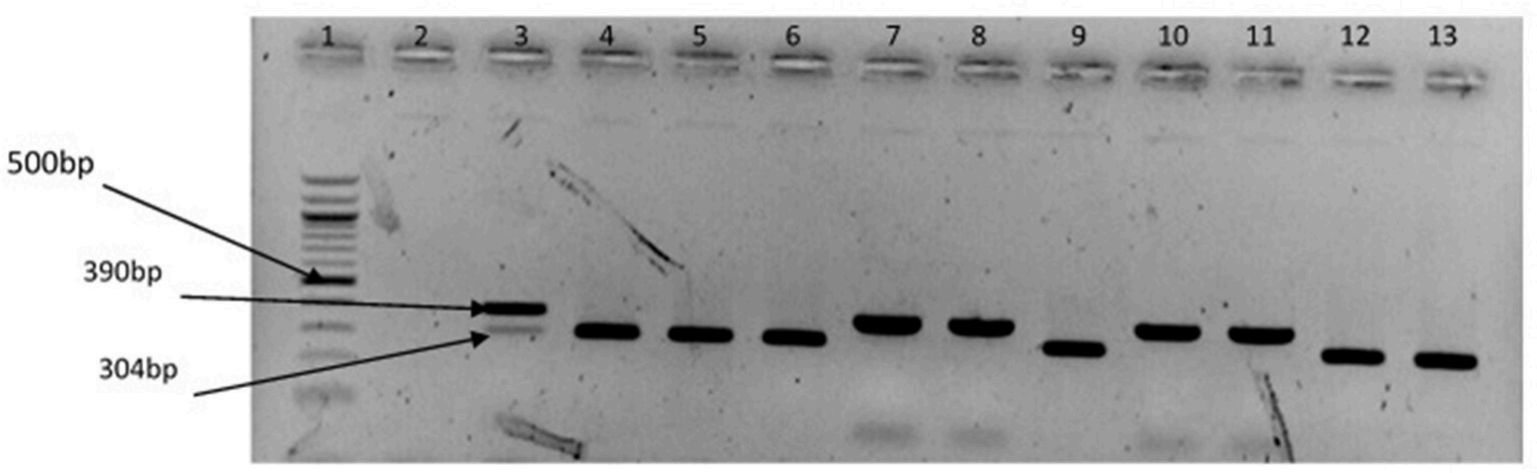
Duplex PCR products of the amplification of OmpW and vhm genes regions specific for *V. cholerae* and *V. mimicus*, respectively. Lane 1, molecular weight marker (100 bp); lane 2, negative control; lane 3, positive control (locally isolated *V. cholerae* and DSM 19130); lanes 4–6, 9, 12 &13, *V. cholerae* positive isolates; lanes 7 & 8, 10 &11, *V. mimicus* positive isolates.

**Figure 5:**
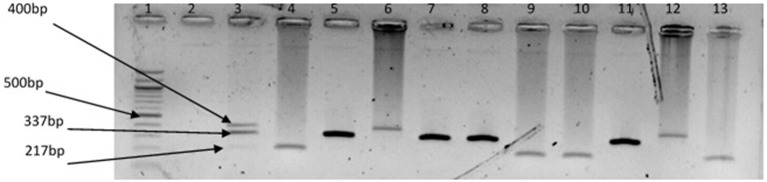
Triplex PCR products of the amplification of GroEl, ToxR, and GyrB genes regions specific for *V. vulnificus, V. fluvialis*, and *V. alginolyticus*, respectively. Lane 1, molecular weight marker (100 bp); lane 2, negative control; lane 3, positive control (DSM 19130, DSM 10143, DSM 19283); lanes 4, 9 &10, 13, *V. fluvialis* positive isolates; lanes 5, 7 & 8, 11, *V. alginolyticus* positive isolates; lanes 6 &12, *V. vulnificus* positive isolates.

**Figure 6:**
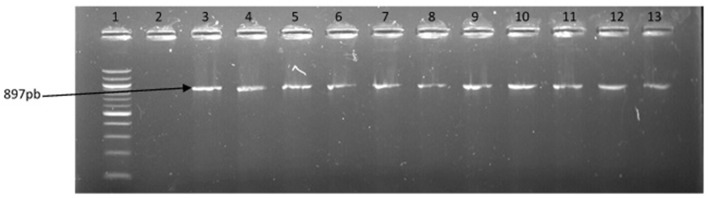
PCR products of the amplification of a region of fla E gene specific for *V. parahaemolyticus*. Lane 1, molecular weight marker (100 bp); lane 2, negative control; lane 3, positive control; lanes 4–13, positive isolates.

The representative samples of electrophoresis gel pictures of PCR product of the amplification of *Vibrio* genus-specific region of the 16S rRNA gene is as shown in Figure 3 while electrophoresis gel pictures shown in Figures 4, 5 are for duplex PCR amplicons for the confirmation of *V. cholerae*, and *V. mimicus*; triplex PCR for the confirmation of *V. alginolyticus, V. fluvialis*, and *V. vulnificus*, respectively. An electrophoresis gel picture of the singleplex PCR product for the confirmation of *V. parahaemolyticus* is given in Figure 6.

## Discussion and conclusion

Halophilic bacteria are a group of organism that has the ability to survive in the hypersaline environment (38). The occurrence of this group of bacteria and their involvement in human infections and the industrial application have been studied over the years (39–41). As an example, (42) reported a more cost-effective application of halophile in the bio-treatment of saline wastewater while gastroenteritis outbreak linked to halophile has been reported as far back as 1958 by Takikawa, in Yokohama, Japan. Although the occurrence of halophile in saline water has been well documented in the literature, the uniqueness of the finding of this study lies in the fact that halophile's density in limpet samples was significantly higher than that observed in the source water samples at the three sampling points (Table 3). A similar observation was made for *Vibrio* spp. except for the density of the bacteria in limpet samples and source water samples from site EL5 which were not significantly different (Table 3). This observation showed that limpet has the potential to concentrate halophile especially *Vibrio* species some of which have been implicated in various human infections such as cholera, cholera-like infections, wound infections, and gastroenteritis. The isolation of five (*V. cholerae, V. fluvialis, V. alginolyticus, V. vulnificus, V. parahaemolyticus*) out of the six key *Vibrio* pathogens investigated in this study from the limpet samples further confirmed the aforementioned. Literally, Figure 2 showed that the five *Vibrio* pathogens were more prevalent in limpet samples than they are in source water samples but this was only significant for *V. parahaemolyticus* and *V. alginolyticus*. This observation suggests that while limpets found in the sampling sites could serve as maintenance reservoir for *V. parahaemolyticus* and *V. alginolyticus*, they could serve as transient reservoir for *V. cholerae, V. fluvialis*, and *V. vulnificus*. These findings explain to an extent while *V. parahaemolyticus* and *V. alginolyticus* are the most reported etiological agents of foodborne infections associated with seafood (43). Interestingly, *V. vulnificus* was only isolated from limpet samples while *V. mimicus* was isolated from only the source water samples. This observation suggests that *V. vulnificus* in the aquatic environment studied better adapt and proliferate within limpet than in the limpet source water where they might be existing in the viable but nonculturable state (VBNC). Ability of *V. vulnificus* which is responsible for 95% of all seafood-associated fatality in the United States (44), to undergo viable but nonculturable state at slightest of unfavorable conditions in an attempt to withstands a whole lot of stress that could occur in aquatic milieu, has been severally reported (45, 46). The fact that limpet source water studied serves as receiving watershed for industrial final effluents, run-offs and different types of wastewater which when poorly treated becomes toxic to aquatic ecology (47–49) supports our assertion. On the other hand, the isolation of *V. mimicus* which was only in source water samples suggests that the limpet recovered from the sampling points are not concentrating the organism. Although the presence of virulence determinants in the six *Vibrio* species focused on in this study was not consider here, the fact that vibrio easily undergo inter and intragenic exchange of genetic materials via horizontal and gene transfer (50–52) makes them potentially dangerous pathogens which if manage to enter human population, could cause serious vibrio infection outbreaks. Furthermore, the distribution of the *Vibrio* spp. targeted in this study per season (Table 4) suggests that vibrio related infections will be common during warm season of the year in our sampling area. Outbreaks of vibrio infections commonly occur in the warm months of the year (53) therefore public health officers should be on a lookout for vibrio infections outbreaks during this period. The general populace need to be careful in the course of their recreational activities during this period as well.

It is worthy of note that the mollusk herein studied is at the lower trophic level of the aquatic food chain and thus will definitely contribute significantly to the occurrence, distribution and prevalence of any organism it is concentrating in aquatic animals such as crab, fish, water birds etc. that consume it at the higher trophic level of the food chain due to the process called biomagnification. Moreover, the finding of this study is of importance to public health and ecology of the aquatic environment since limpet serves as seafood recipe for human and an important member of the lower trophic level of the aquatic food chain (13, 54). Foodborne *Vibrio* infection outbreaks caused by species of *Vibrio* isolated from limpet samples processed in this study have been reported (55–60). It is important to note that cooking limpet at high temperature for 30 sec or at low temperature as recommended by Fitzgerald (11) will increase chances of contracting vibrio infection from vibrio contaminated limpet. This is because the cooking temperature is either sub-leather to the pathogens or the cooking duration is not long enough to achieve total destruction of all *Vibrio* pathogen cells that may be present in the limpet.

Therefore, in the best interest of the public health and aquatic ecology, we recommend more studies on the role that limpet, an abundant mollusc in South Africa aquatic environment, plays in the maintenance of pathogens in the aquatic milieu. It is anticipated that the information from such studies will shed more light on other possible diseases outbreaks aside cholera and vibriosis, which could emanate from the consumption of limpet recipe.

Finally, this is the first study that attempts differentiating between transient and maintenance reservoir of pathogens by employing the combination of MPN-PCR method and statistical analysis. Furthermore, although isolation of key *Vibrio* pathogens from various types of mollusc has been severally reported (5, 19, 31, 61–63), to the best of our knowledge no report has implicated limpet. Since a comparative high abundance of *Vibrio* spp. which was statistically significant was observed in limpet than in source water in this study, and that the five of the six *Vibrio* pathogens targeted in this study were more prevalent in limpet than in source water samples, we concluded that limpets found in the estuaries are not just a transient reservoirs but a maintenance reservoirs for *Vibrio* species.

## Ethics statement

The animal samples used in this study were invertebrates from the wild and the samples were purchased from fishermen. Also, the quantity sampled per time (10–15 pieces) could not be considered to affect the population of organism in the wild and limpet (Scutellastra cochlear) is not an endangered species.

## Author contributions

AO initiated the research topic, provided materials for the study, and proof-read the manuscript. OA structured the methods used in the study, carried out the experiments, and write the manuscript.

### Conflict of interest statement

The authors declare that the research was conducted in the absence of any commercial or financial relationships that could be construed as a potential conflict of interest.
